# Dr. Walker, on the Eye

**Published:** 1807-06

**Authors:** John Walker

**Affiliations:** Salisbury Court


### To the Editors of the Medical and Physical Journal.

Gentlemen,

On "one entrance" to wisdom, to the poet shut up as he sings in moving strains, I offer you a most hasty sketch of a few simple ideas. They were excited in me a few days ago, during the discussion of a paper on Strabism. I am of opinion, that the only cause of irremediable squinting, is a distortion, greater or less, or a position more or less oblique, of the crystalline lens of one eye, when it is not of both. In such case of obliquity of one eye, the focal point will not fall, as in the other perfectly formed one, on the part of the retina, directly opposite to the pupil; the optical axis will not be the longest possible line across the eye, or the whole diameter of it; the central ray, from the *point* of vision, *regarded,* will not strike directly the summit of the cornea, as in the eye, with all its parts, correctly arranged; (I now for simplicity's sake, debarrass myself myself of the consideration of refraction) the ray will fall obliquely on one side of the apical point of the cornea. The eye must always have a distorted appearance to an observer.

Under such derangement of the position of the whole organ, as must be produced by any obliquity in the position of the crystalline, can the vision be so perfect as in the perfectly formed eye? The resources of nature are wonderful. There will be a great accommodation of parts to the obliquely placed crystalline, which will, perhaps, even be brought forward, that its distance from the focal point, on the retina, may be the same as in the perfect eye; in fact, that the focal point may not fall behind, or without the retina. Yet, though the *anatomically* non-corresponding points of the two eyes may be possessed of equal sensibility, and may be perfect *optically* corresponding points, producing nothing like double vision, perhaps the direct eye will always, *ceteris paribus*, have more perfect vision than the oblique one.

I have spoken of irremediable strabism; because, I believe a habit of squinting may be acquired, where there has not been an original distortion, or obliquity, of the lens. I remember myself, when a school-boy, to have so much acquired the habit of seeing things double, when I wished so to amuse myself, that, at length, it sometimes occurred to me involuntarily; and I have known others who have felt it both troublesome and painful. Our method of getting rid of the dazzling illusion, was by turning from the particular objects of vision at the time, and thus, in some way, bringing the optical axes of the eyes so to converge upon others, as to restore to us our single vision. Now, as it happens, I believe with most people, that either from habit, (a habit insensibly acquired in our acquiring by experience single vision of the objects all around us) or from original difference in the two eyes, they see rather better with one eye than with the other; perhaps, in all the playful instances of squinting, we commonly see the objects of attention more distinctly with the good eye, than with the weak one, which only takes a very vacant kind of notice of them; and, perhaps, if the distortion were sufficiently long continued, other points than the anatomically corresponding points of vision, would, at length, become the optically corresponding points. The weak eye would yield, or accommodate itself to the superior attention of the other; and distorted in its socket, it would roll about consentaneously, with it, constantly receiving the picture of the objects of vision, on a different part of its retina, from what it had done in the beginning; but, producing, at length, single vision as completely as before the distortion took place.

In such cases of strabism, and, perhaps, in such only, may we expect to remove the appearance of squinting, by means of art, by causing the weak eye, that has wandered, to take its former and natural situation. A small tube, fixed directly before the centre of such eye, confining it to looking only directly forward, would, perhaps, by degrees, at length restore it to its 'proper bearings.'

On whatever part of the Creation we turn our eyes, the laws we find written there, as far as we can comprehend them, may make us exclaim with the philosophic orator of Rome.—*Doceat ergo aliquis potuisse melius: sed nemo nunquam docebit ut si quis corrigere aliquid volet, aut deterius faciet, aut id quod fieri non potest, desiderabit.*—Marc. Tul. Cicero. I am led to the above observation, from the persuasion, that in the structure and disposition of the eyes, there is evidently a very simple provision, for generally preventing strabism, in the object of direct vision making the strongest impressions on the retinæ: moreover, it is most natural and easy for us to turn the whole countenance, as well as to direct the eyes to the object we wish to behold most stedfastly.

In the newly-born infant, feeling the stimulus of the flood of light pouring into its opening eyes, new sensations are excited by the impressions of the rays on its retinæ. The passive organs of vision, by their structure, lie even, if we may so express it, in their sockets. The points of the retinæ, directly opposite the pupils, are anatomically the same, or corresponding, points. A star shedding its rays on the countenance of the babe, would produce images, or impressions of itself on these points; or, if without them, infinitely near to them. The eyes being perfectly formed, the crystalline lenses are well adapted to make the object so fall, or to make the corresponding points become the focal points of direct vision. While we readily imagine there will naturally be a sympathy the most acute, between these corresponding nervous points; we find that this is not confined to the only two points of anatomical correspondence. In regarding any object attentively, suppose the letter B, the straight side of the letter will, in the left eye, be within such point; the bowed side without it: exactly the contrary disposal of it, in the right right eye, will take place; yet, confusion does not ensue, we see the B as one single character. At the same time it is observable, that even in the smallest letter, we can far more intensely look at any particular point of it, than at the whole character; and the child very soon, by the sympathy between the correspondent points, gets to turning its eyes inward, e. g. on a candle shining upon its countenance, in order to bring the impressions of the flame, seen under an infinitely larger angle than the star, on the corresponding points of the retina. This most intimate sympathy, however, between the corresponding nervous points, evidently depends upon their habitually acting consentaneously, as the focal points of direct vision do not ever correspond anatomically in squinting persons.

When the focal points of the object of direct vision fall on the corresponding spots of the retinæ, which, in perfectly formed eyes, they naturally do, the organs accommodating themselves to the distance of the object of view, not only by their optical axes converging on the object, but also by minute consentaneous change of form in their transmitting media, like unto so many lenses; then the most vivid impression is produced on those consentaneously stimulated, ever sympathising, spots. We have learnt by experience, that the two impressions are produced by one object. By an abstraction from the first sensible effects on the two retinæ, the mind recognises them as one; and the idea of unity alone is thus excited by the presence of one object. At such time, we have a vacant sort of sense of single vision, or perception, of the things in view all about us, gained also from experience. When the object of direct vision is thus vividly painted on the corresponding spots of the two retinæ, the focal points of the objects within or beyond it, in the same direction, fall not exactly on the same spots. They fall within or beyond the retinæ. Their impression on these nervous fields is very faint and obscure; but though the perception of them is faint and obscure, yet, if we bring our attention to bear on them distinctly, we shall find that they do actually produce in us, double vision. If looking attentively at a distant object, I hold a finger up before me, the finger appears as two; if the eyes be brought to bear directly on the finger, it no longer is seen as two, but the distant object appears double.

Do not these observations also apply to near and distant objects, more or less remote from the line of direct vision? They would certainly apply; the foci of such objects would be found within or beyond the retina. Such objects would appear both obscure and double; but when it is considered that objects out of the line of direct vision, though at proper focal distances, hereby making distinct images on the retinæ, *(par parenthese,* no object could be exactly at a proper focal distance from each eye, except in the line of direct vision, or vertically only [vertex capitis] above or below it) are only objects of a very vacant kind of notice to us; that the optically corresponding points on the retinæ of such objects, even, continually change or vary, as the distance of our object of direct vision, varies its distance; perhaps, they also constantly produce in us a kind of double vision, and the faint confusion arising from these things, we perhaps get rid of, not only by one eye constantly taking the lead, if I may so express it in observation, but also by the mind or attention being so directly fixed, only, on the object of direct vision, that it very little heedeth these indistinct and oblique parts of the great field of vision before us.

Perfectly formed eyes have little inducement to wander into habits of strabism.

It is most natural to us, to look directly forward; we hereby obtain the most perfect vision. When we have turned our eyes, without moving the head to an object, on one side, it is at different distances from the two eyes, and consequently seen under different angles by them. Perhaps the two optic nerves are not then in their most natural positions; their condition, in point of tension, must be considerably different. These remarks do not apply to the action of throwing the eyes upwards or downwards, without moving the head; and the vertical field of distinct vision seems more extensive than the horizontal one.

JOHN WALKER.

*Salisbury Court,* 17 *iv,* 1807.

P. S. Will you add, for the information of numbers of your readers, whom I have had the pleasure of supplying during the last four years with Vaccine Ichor, and which I always select myself, that if they address "Dr. Walker, London Vaccine Institution," their applications will be immediately attended to. But as the privilege of having the correspondence franked, has been taken from every Society, it will be proper that they remember to pay the postage; otherwise the Institution will continue to be put to a considerable daily expence.

